# Digital twin technologies in prostate cancer as a frontier for precision medicine

**DOI:** 10.1186/s41747-026-00753-8

**Published:** 2026-06-19

**Authors:** Martina Pecoraro, Emanuele Messina, Simone Novelli, Ludovica Laschena, Graziano Blasilli, Enrico Tronci, Valeria Panebianco

**Affiliations:** 1 Department of Radiological Sciences, Oncology and Pathology, Sapienza University of Rome, Rome, Italy; 2 Department of Mechanical and Aerospace Engineering, Sapienza University of Rome, Rome, Italy; 3 Department of Computer, Control and Management Engineering, Sapienza University of Rome, Rome, Italy; 4 Department of Computer Science, Sapienza University of Rome, Rome, Italy

**Keywords:** Artificial intelligence, Computer simulation, Overtreatment, Precision medicine, Prostatic neoplasms

## Abstract

**Abstract:**

Prostate cancer (PCa) is the most frequently diagnosed malignancy among men and presents major clinical and socioeconomic challenges worldwide. Despite advances in early detection, imaging, and therapy, managing PCa remains complex due to disease heterogeneity, risks of overdiagnosis, and overtreatment. Digital twin (DT) technologies might represent an emerging conceptual framework aimed at supporting dynamic, patient-specific virtual modeling for personalized clinical decision-making. By integrating multimodal clinical, imaging, molecular, and physiological data, DTs can simulate disease progression, predict treatment responses, and support proactive, adaptive care. This perspective explores the conceptual framework for DT ecosystems in PCa, highlighting potential clinical impacts, infrastructural requirements, and barriers to implementation. Harnessing DTs could impact PCa management into a truly predictive, personalized, and participatory approach, improving outcomes and optimizing healthcare resource utilization globally.

**Relevance statement:**

DT technologies may enable personalized, predictive PCa management by integrating multimodal patient data to guide diagnosis, treatment selection, and monitoring, with the potential to improve outcomes, reduce overtreatment, and optimize healthcare resource utilization

**Key Points:**

- DTs create virtual patient models to personalize PCa care.
- They integrate imaging, clinical, and molecular data into one system.
- This approach may reduce overdiagnosis and unnecessary treatments.

**Graphical Abstract:**

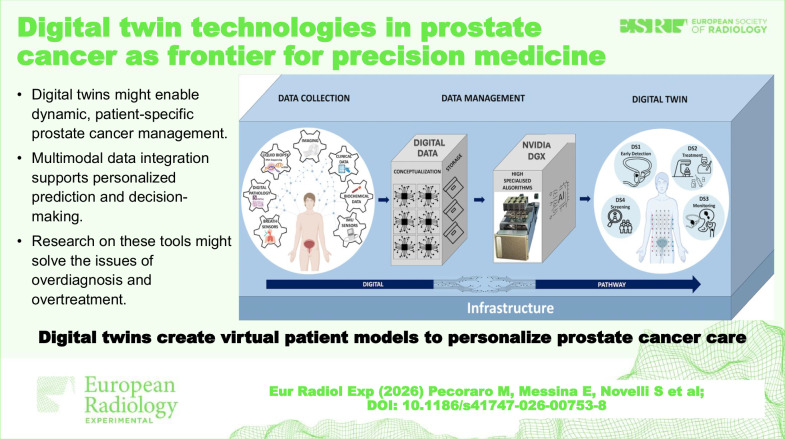

## Background

Prostate cancer (PCa) represents a clinical and public health challenge, affecting millions of men worldwide. According to recent epidemiological estimates, the global burden of PCa is expected to rise sharply in the coming decades, with projections indicating an increase from approximately 1.47 million new cases in 2022 to over 2.6 million cases by 2045 [1]. This alarming trend reflects a combination of factors, including population aging, improved awareness and screening practices, as well as limitations of current diagnostic paradigms, which may contribute to overdiagnosis and the detection of clinically indolent tumors that might not otherwise become symptomatic.

PCa is a highly heterogeneous disease. Its biological behavior can vary dramatically, ranging from indolent, slow-growing tumors that may never pose a significant threat to life, to aggressive forms that metastasize rapidly and result in substantial morbidity and mortality [2–5]. This heterogeneity poses a central dilemma for clinicians: how to distinguish between cases that require prompt, aggressive treatment and those that can be safely managed with active surveillance or conservative approaches. Screening strategies, predominantly based on serum prostate-specific antigen (PSA) testing, have undoubtedly contributed to earlier detection and reduced mortality rates [6, 7]. However, PSA testing lacks specificity and is associated with high rates of false positives, leading to unnecessary biopsies and overtreatment of clinically insignificant cancers [7, 8]. In addition to total PSA levels, PSA-derived biomarkers such as PSA density are commonly used to refine risk stratification, improve specificity, and guide decisions regarding biopsy and surveillance. While multiparametric magnetic resonance imaging (mpMRI) and the prostate imaging-reporting and data system (PI-RADS) score have emerged as a powerful tool for improving diagnostic accuracy and enabling targeted biopsies, it is not without limitations [2, 9]. Inter-reader variability, limited availability in many healthcare systems, and costs restrict its widespread use, particularly in low- and middle-income countries, where the burden of late-stage disease remains substantial [4].

Indeed, currently, the diagnostic pathway for early disease diagnosis in high-income countries mainly relies on MRI and has been associated with substantial benefits. MRI-based diagnosis has high negative predictive value and sensitivity for clinically significant cancers, which enables reliable ruling out of clinically insignificant cancers without the need for biopsies [10, 11]. The use of MRI has led to the definition of “MRI pathway” that, with MRI-directed biopsy, enables the delivery of key diagnostic benefits to men suspected of having cancer according to their clinical priorities [12, 13]. On the contrary, MRI-driven workflows remain susceptible to low specificity and high inter-reader variability [14].

Beyond the initial diagnosis, the complexity of treatment decision-making in PCa is equally daunting. Clinicians must balance the risks and benefits of different therapeutic options, taking into account tumor characteristics, patient comorbidities, life expectancy, and personal preferences [2]. The decision to pursue radical prostatectomy, radiation therapy, or active surveillance is fraught with uncertainty and carries significant implications for patient quality of life, especially given the potential for urinary, sexual, and bowel dysfunction associated with definitive treatments [15, 16].

Beyond initial diagnosis and primary treatment selection, several additional clinical scenarios remain highly dependent on imaging and longitudinal risk assessment [17]. These include biochemical recurrence after definitive therapy, management of locally advanced or recurrent disease, non-metastatic castration-resistant PCa, and the differentiation between oligometastatic and polymetastatic states [18–21]. In addition, recent advances in systemic therapies, including androgen receptor pathway inhibitors and novel radiopharmaceuticals, have improved outcomes but also introduced complexities related to sequencing, patient selection, and monitoring response [2]. In these settings, repeated imaging, quantitative biomarkers, and dynamic disease modeling may be particularly suited to “digital twin” (DT) approaches, supporting treatment adaptation and personalized monitoring throughout the disease course.

In this multifaceted landscape, there is a growing consensus that traditional, one-size-fits-all approaches are insufficient. There is an urgent need for tools capable of integrating diverse clinical, molecular, and imaging data to provide dynamic, personalized insights that can guide decision-making throughout the disease course. This is precisely the promise offered by DT technology, an innovative concept that seeks to bridge the gap between complex biological realities and actionable clinical strategies.

The aim of this narrative review is to critically describe the current state of DT research in PCa, discuss potential clinical applications across the disease continuum, and outline the technical, methodological, and organizational challenges that must be addressed before these approaches can be reliably translated into routine care. Unlike prior systematic reviews that primarily summarize and benchmark computational DT models, this narrative review adopts a translational and radiology-focused perspective, emphasizing imaging integration, clinical workflows, and infrastructural considerations necessary for real-world implementation.

## The promise of DT technologies for PCa

The concept of a DT originates from engineering and manufacturing, where systems are often deterministic and well characterized, and it refers to the creation of a precise virtual replica of a physical object or system [22]. This digital model can be continuously updated with data from the real-world counterpart, allowing for simulations, predictive analyses, and performance optimization [22]. Its application to healthcare and oncology represents a significant extrapolation, as biological systems are inherently complex, variable, and only partially understood. Consequently, current DT models in medicine should be regarded as approximate and probabilistic representations rather than exact replicas, with their clinical reliability and precision still requiring rigorous validation.

In practical terms, a DT can be defined as a continuously updated computational model that replicates the clinical and biological state of an individual patient by integrating multimodal data, including clinical variables, imaging findings, pathology, and molecular information, into a unified analytical framework. Operationally, this process involves four key steps: (1) systematic data acquisition; (2) construction of predictive or simulation models using artificial intelligence (AI) and statistical methods; (3) generation of individualized risk estimates for disease early detection or treatment-response predictions; and (4) iterative updating of the model as new patient data become available [23–26]. In clinical practice, such a framework may function as a decision-support tool, providing personalized forecasts to assist clinicians in diagnosis, treatment selection, and longitudinal monitoring [27].

However, even though in healthcare systems DT technology has been utilized to build digital representations of healthcare processes, building the DT simulating a single individual/patient is currently still difficult [22, 28]. Indeed, the knowledge of the complexity of the molecular events underlying the human body physiology, the pathogenesis and progression of the thousands of different recognized diseases, and the mechanisms of action of many drugs, are very far from being fully understood; thus, any modeling will be highly imprecise [23, 29]. On the other hand, populations of patients (clusters), homogeneous with respect to a specific outcome in each disease stage, may represent a feasible model exploitable for DT simulations, for comprehensive profiling of physiological and pathological states. The application of DT in healthcare holds extraordinary potential. Rather than relying solely on static clinical assessments, a DT framework integrates longitudinal patient data to enable dynamic and personalized predictions [30]. This virtual twin would integrate longitudinal data from electronic health records, high-resolution imaging studies, genomic, molecular, and proteomic profiles, and even data from biosensors capturing physiological and biomechanical signals. By continuously updating and recalibrating, the DT could simulate how a patient’s cancer might progress, how it would likely respond to different treatment modalities, and what complications might arise along the way.

Indeed, it is important to underline that DT differs from a conventional electronic health record or data repository. While an electronic health record aggregates and stores heterogeneous patient data, it remains primarily descriptive and retrospective. In contrast, a DT incorporates these data into computational and predictive models that dynamically simulate disease behavior, estimate future outcomes, and evaluate hypothetical treatment scenarios. Thus, the DT represents not merely a digital copy of patient information, but an active decision-support framework built upon modeling and simulation capabilities.

Such a model would enable truly personalized medicine, moving beyond population-based risk calculators and guidelines to a more patient-centric approach. Decisions about whether to biopsy a suspicious lesion, when to initiate or escalate therapy, or how to balance oncologic control with quality-of-life considerations could all be informed by individualized predictions generated from the DT. Furthermore, DT technology could impact the monitoring of treatment responses and disease progression. In advanced PCa, where therapeutic resistance and metastatic spread are major concerns, a DT has been proposed as a potential tool to investigate whether earlier indicators of disease progression could be identified. In addition, by modeling various treatment scenarios *in silico* before actual implementation, clinicians could better anticipate side effects and optimize therapeutic regimens, ultimately enhancing patient outcomes and reducing healthcare costs. Nonetheless, the integration of DT into routine clinical practice faces significant challenges. These include technical hurdles related to data integration and standardization, the need for robust and transparent AI algorithms capable of handling complex, multimodal data, and ethical considerations surrounding patient privacy and data security [30]. Moreover, clinical validation through large-scale prospective studies will be essential to establish credibility and gain the trust of both clinicians and patients.

The potential clinical applications of DT technology across different disease settings in PCa are summarized in Table 1.

**Table 1:** Clinical applications of DT technology in PCa

Disease setting	Clinical focus	DT application	Expected benefit
1	Early diagnosis	Simulation model for detecting clinically significant PCa	Timely, accurate diagnosis; avoid unnecessary biopsies
2	Treatment decision-making	Decision support system simulating outcomes for different treatment options	Personalized therapies, reduced complications, improved quality of life
3	Monitoring of advanced disease	Real-time monitoring system for advanced and metastatic PCa	Early identification of progression, optimized therapy adjustments
4	Risk stratification and screening	Predictive simulation to identify at-risk populations	Proactive screening, reduced mortality, and overtreatment

*DT* Digital twin, *PCa* Prostate cancer

## Toward a DT ecosystem for PCa

As previously mentioned, the implementation of DT technologies requires dedicated digital infrastructures capable of integrating heterogeneous clinical, imaging, pathology, and molecular data into interoperable platforms. Such systems must combine secure data repositories, standardized formats, high-performance computing, and embedded predictive models to enable real-time analysis and decision support. In this context, the DT functions not as an isolated application but as a coordinated ecosystem linking hospital information systems, imaging archives, and analytical tools within routine clinical workflows.

### Integrated diagnostics digital data

Clinical data represent the backbone of any DT framework in PCa, and their systematic integration remains a critical requirement. Anonymized patient information, including demographics, medical history, vital signs, physical examination findings, laboratory test results (particularly PSA levels), medications, treatment plans, lifestyle and social history, allergies and adverse reactions, mental health data, procedures, interventions, and patient-reported outcomes such as the International Prostate Symptom Score questionnaire, should be captured using standardized electronic case report forms. To ensure reproducibility and interoperability, the use of subject identifiers and trial numbers has been widely recommended, with clear communication of data usage outlined in participant information materials. Importantly, responsibility for ensuring data accuracy and completeness is generally emphasized as a key role of clinical investigators.

Diagnostic imaging constitutes another fundamental layer in PCa digital ecosystems. Qualitative and quantitative data extracted from mpMRI, computed tomography (CT), positron emission tomography (PET) with radiotracers such as ^68^Ga-prostate‑specific membrane antigen (PSMA), bone scans, and transrectal ultrasound are routinely considered essential inputs for staging, diagnosis, and longitudinal monitoring. The integration of these imaging modalities into unified infrastructures facilitates multimodal assessment and lays the groundwork for advanced modeling and simulation.

Similarly, digital pathology has been increasingly recognized as an innovative diagnostic tool. Computer-driven pathology assessment and virtual microscopy enable rapid access to archived images, faster turnaround of complex cases, and improved opportunities for collaborative diagnostics. Histological specimens digitized through high-resolution scanners can be shared via secure online servers, thus supporting remote consultation and multidisciplinary evaluation. Such infrastructures are widely considered necessary to scale precision oncology approaches and integrate pathology into DT environments.

Finally, “omics” data are also gaining prominence in this context. Both liquid and tissue biopsies provide material for high-throughput analyses, including whole-genome single-nucleotide polymorphism (SNP) genotyping to assess heritable predispositions and gene–environment interactions, as well as microribonucleic acid (microRNA) expression profiling from plasma or MRI-targeted tumor biopsies. These molecular datasets help to define the biology of clinically significant PCa and identify candidate biomarkers, such as microRNAs, driving disease progression. Standard operating procedures are crucial to ensure quality, reproducibility, and comparability across institutions.

**Fig. 1:**
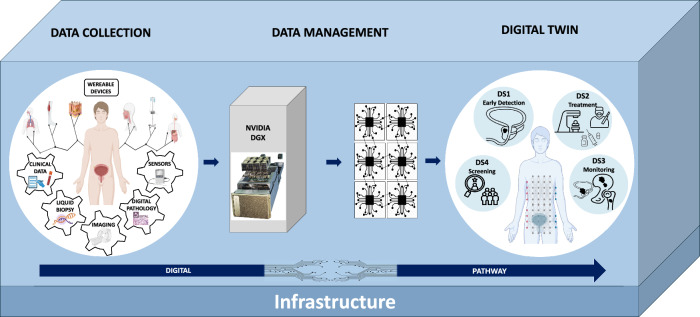
*Schematic* representation of the data flow for DT development in PCa care. The architecture is presented as a generic DT framework that could be adapted to different diseases; PCa is used here as an illustrative clinical use case. The figure was created with BioRender (www.biorender.com)

**Fig. 2:**
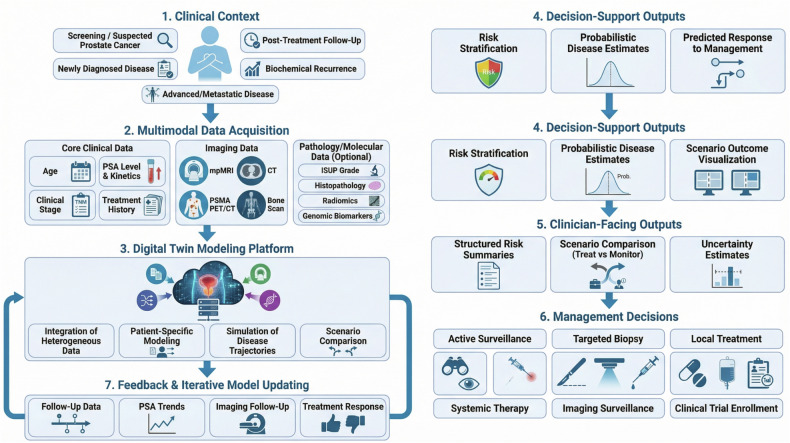
Conceptual workflow illustrating how DT frameworks may support clinical decision-making across different PCa disease settings. Multimodal clinical, imaging, and pathology data are integrated into a patient-specific modeling platform to generate probabilistic, scenario-based decision-support outputs. These outputs are intended to assist clinicians in risk stratification and management selection, without replacing clinical judgment. The figure represents a generalized and illustrative framework rather than a validated clinical system. The figure was created with BioRender (www.biorender.com)

Taken together, these diverse datasets highlight the scope of information streams that could be integrated into comprehensive DT infrastructures. Clinical, imaging, pathology, and molecular data each contribute distinct but complementary layers of information. Future review and research efforts should continue to focus on how these multimodal sources can be harmonized within secure, interoperable, and scalable infrastructures to advance precision oncology and personalized PCa care. Figure 1 summarizes the data flow, while Fig. 2 illustrates the conceptual workflow on how DT frameworks may support clinical decision-making across different PCa disease settings.

### Infrastructure for DT development

**Fig. 3:**
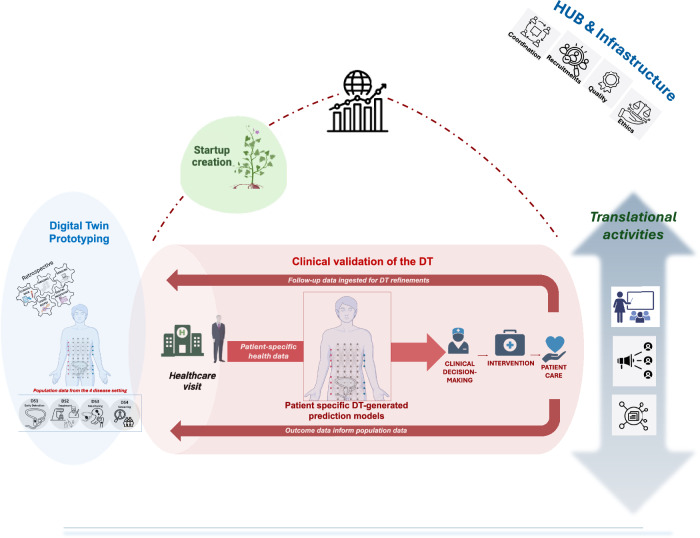
Workflow for the development, validation, and translation of a DT in healthcare. The process begins with DT prototyping (left), where population-level data from different disease settings (omics, imaging, clinical, etc.) are integrated to generate initial DT models. During the healthcare visit, patient-specific health data are incorporated into the DT to produce individualized prediction models. These models support clinical decision-making, guide interventions, and improve patient care. Follow-up data are continuously fed back into the DT for refinement, ensuring iterative learning and model improvement. The cycle of patient-specific outcomes also informs population-level datasets, creating a dynamic loop between individual and collective knowledge. The DT clinical validation pipeline (central image) is supported by HUB and infrastructure (top right), which ensures coordination, resources, quality, and ethical oversight. Translational activities (right) include training, dissemination, and clinical adoption, promoting the integration of DT-based decision support into practice. Finally, the process fosters startup creation (top left) and innovation pathways, translating research outputs into practical healthcare solutions. The diagram is conceptual and intended to illustrate potential translational pathways rather than to imply automatic implementation or guaranteed success. The figure was created with BioRender (www.biorender.com)

The realization of a comprehensive DT ecosystem in PCa care fundamentally relies on robust, scalable, and interoperable infrastructures [24]. Developing such infrastructures requires seamless integration of diverse data types into a unified, secure, and standardized framework [25, 26]. A dedicated digital hub should be envisioned to serve as the core operational environment, enabling real-time data storage, processing, and simulation. This digital backbone must be supported by high-performance computing capabilities to handle large volumes of multimodal data and perform complex predictive modeling at scale. Standardized data formats and interoperability frameworks are critical to ensure consistency and facilitate integration across different institutions and healthcare systems, both locally and globally. Additionally, strict adherence to data governance and privacy standards is essential, incorporating advanced security protocols to protect sensitive patient information and foster trust among stakeholders. Beyond technological elements, the infrastructure should support continuous learning and iterative refinement of models, leveraging AI approaches to update predictive algorithms without compromising data privacy. Importantly, this infrastructure should be designed to be adaptable and equitable, enabling deployment in diverse clinical contexts, including resource-limited settings. By providing this robust operational backbone, digital infrastructures will be key to transforming the conceptual promise of DT technology into practical, clinically actionable tools that support personalized, predictive, and participatory PCa care. Figure 3 shows the workflow for the development, validation, and translation of a DT in healthcare. A summary of the core technical and operational requirements needed to establish a scalable and interoperable DT ecosystem in PCa is provided in Table 2, outlining the essential infrastructural components and their functions.

**Table 2:** Key technical and operational requirements for a DT ecosystem

Infrastructure component	Description
Data integration layer	Unified platform for clinical, imaging, omics, pathology, and any data belonging to patients
High-performance computing	Real-time processing and simulations across thousands of concurrent users
AI algorithms	Multimodal deep learning and causal inference models for prediction and simulation
Interoperability standards	Compliance with HL7/FHIR, GDPR, and medical imaging formats for global scalability
User interface	Clinician-friendly dashboard with predictive outputs, alerts, and treatment scenario explorers
Security framework	End-to-end encryption, access control, and ethical oversight for data protection

*DT* Digital twin*, FHIR* Fast healthcare interoperability resources, *GDPR* General data protection regulation, *HL7* Health Level Seven International

### What has already been done and what is hypothesized

Recent advances have reinforced the potential of DT technology in PCa care. According to a systematic review by John et al [31], reported that DT-oriented approaches integrating multimodal inputs (*e.g*., clinical variables, imaging/radiomic features, and molecular data) were generally associated with improved performance for clinically relevant tasks such as detection/risk stratification and prediction of outcomes (*e.g*., progression or recurrence), compared with single-modality models; however, performance metrics and comparators varied substantially across studies. Meanwhile, Stamatakos et al [32] introduced a multiscale simulation model that supports *in silico* treatment evaluation, laying the groundwork for interactive digital consultations. These findings collectively validate the DT paradigm not only as a technological innovation but as a critical enabler of participatory, patient-centered oncology.

Also, Camacho-Gomez et al [27] proposed a physics-informed machine learning DT to reconstruct PCa tumor growth from serial PSA tests. Patient-specific DTs were derived from multiparametric MRI (T2-weighted, diffusion-weighted, dynamic contrast-enhanced sequences) to integrate anatomical, vascular, and cellular features, while a physics-based model simulated PSA secretion and tumor proliferation. A deep neural network regulated growth trajectories by combining PSA blood tests with spatial physiological data. In a limited proof-of-concept analysis involving two clinical cases, the framework was able to reproduce observed tumor evolution patterns, illustrating the technical feasibility of integrating PSA kinetics with imaging-derived features [27]. This approach improves the interpretability of PSA monitoring and may reduce the need for repeated MRI. However, a key limitation is that the framework does not fully integrate all available longitudinal data from individual patients, which may restrict its generalizability. Also, these preliminary findings are exploratory and do not constitute evidence of clinical effectiveness.

Finally, Kim et al [33] developed a machine learning–based DT system to predict PCa progression, specifically focusing on pathological staging and biochemical recurrence. Using data from 404 patients extracted from a clinical data warehouse, the authors trained multiple machine learning models (logistic regression, random forest, SVM, neural networks, etc.) to sequentially predict pathology outcomes (T stage, Gleason score, extracapsular extension, seminal vesicle invasion, lymph node status, etc.) and subsequent biochemical recurrence. The DT framework linked real and virtual models of the PCa process, allowing predictions to be simulated step-by-step. The best-performing algorithm was the random forest, achieving accuracies up to 98.5% for pathological T stage and 96.3% for biochemical recurrence, demonstrating strong predictive power compared with conventional machine learning approaches [33]. This work highlights the potential of DT-based decision support to improve personalized treatment planning and cost-effective care in PCa. However, a major limitation is that the model was trained on a relatively small and highly selected subset of available patients (404 out of 3,024), excluding incomplete or noisy data. This restricts the generalizability of results and underscores the need for validation in larger, more heterogeneous cohorts.

Realizing this vision requires overcoming substantial challenges, including technical hurdles related to data integration and standardization, the development of transparent and explainable AI algorithms, and the need for rigorous clinical validation. Additionally, patient engagement and education are critical to ensuring that individuals understand and trust their DT, empowering them to actively participate in their care.

## Future perspectives and potential impact

It should be emphasized that, at present, DT technologies in healthcare remain largely exploratory. Most applications are proof-of-concept or pilot studies, and robust prospective validation demonstrating consistent clinical benefit is still limited. Consequently, DTs should be viewed not as fully mature tools but as evolving decision-support frameworks whose implementation will require methodological standardization, regulatory oversight, and careful evaluation of cost-effectiveness before routine adoption

Nonetheless, the integration of DT technology into PCa care heralds a new era in precision oncology.

For patients, this translates into more accurate diagnoses, reduced exposure to unnecessary treatments, and better quality of life through optimized therapeutic pathways. For healthcare systems, DTs offer the potential to improve resource utilization, reduce costs associated with overtreatment and complications, and support more equitable access to advanced cancer care.

However, realizing this potential will require concerted efforts to overcome technical, regulatory, and ethical barriers. The development of transparent, interpretable AI algorithms, robust data governance frameworks, and rigorous clinical validation studies will be critical to ensuring that DTs can be safely and effectively integrated into everyday practice.

### Socioeconomic impact and implications for healthcare systems

The introduction of DT technologies in PCa care carries profound implications not only for individual patient outcomes but also for the broader organization and sustainability of healthcare systems. One of the most pressing challenges faced by modern healthcare is the need to balance rising costs with the demand for increasingly personalized, high-quality care. Overdiagnosis and overtreatment, particularly prevalent in PCa management, contribute significantly to unnecessary healthcare expenditures, patient morbidity, and system inefficiencies.

By providing a highly individualized, predictive approach, DTs offer the potential to dramatically reduce these inefficiencies. Simulating disease progression and treatment responses at the patient level allows clinicians to avoid unnecessary interventions, minimize complications, and better allocate resources to those who are most likely to benefit. This shift from a reactive to a proactive model of care can lead to substantial cost savings by preventing hospitalizations, reducing the need for intensive treatments, and optimizing follow-up strategies.

Moreover, the scalability of DT technologies could help bridge the gap in healthcare delivery between high-resource settings and low- and middle-income countries. In these regions, limited access to specialized diagnostic tools and expert clinical decision-making often results in late diagnoses and poor outcomes. A well-implemented DT framework, once validated and adapted, could support remote risk assessment, guide treatment decisions even in resource-constrained settings, and promote more equitable access to advanced oncological care.

From the perspective of policy-makers, demonstrating the economic value of DT technologies will be essential to secure support from healthcare payers and governmental bodies. Cost-effectiveness analyses, integrated into clinical validation studies, will play a pivotal role in justifying initial investments and driving large-scale adoption. Ultimately, if implemented effectively, DT technologies could become a cornerstone of value-based healthcare models, focusing on optimizing outcomes relative to costs and prioritizing patient-centered care. Table 3 illustrates the potential socioeconomic and healthcare system impacts of DTs, emphasizing their contribution to improved outcomes, cost savings, global health equity, and policy development.

**Table 3:** Socioeconomic and healthcare system impact of DT technology

Impact domain	Contribution of DTs
Clinical outcomes	Personalized risk assessment, optimized therapy, better quality of life
Cost reduction	Avoidance of unnecessary treatments and hospitalizations
Global health equity	Scalable to LMICs; reduces disparity in access to expert decision-making
Healthcare workforce support	Decision support for clinicians, reduces cognitive burden
Policy and planning	Data-driven health policy formulation and resource allocation optimization

*LMICs* Low- and middle-income countries

### Future technological developments and opportunities

Looking ahead, the evolution and maturation of DT technologies in PCa—and more broadly in medicine—will depend heavily on continued technological innovation. One key area of future development is the enhancement of data integration and processing capabilities. As the volume and complexity of healthcare data continue to grow exponentially, leveraging advanced big data analytics and real-time data streaming will be crucial to maintaining the accuracy and relevance of each patient’s digital representation.

The integration of emerging computational paradigms, such as quantum computing, holds the promise of accelerating the speed and scale at which DTs can operate. Quantum algorithms could enable the simulation of highly complex biological processes and treatment scenarios that are currently beyond the reach of classical computing, unlocking new levels of predictive power and personalization.

Another exciting frontier is the incorporation of immersive technologies such as augmented reality and virtual reality. These tools could allow clinicians—and potentially patients themselves—to interact visually and intuitively with their DT, exploring potential treatment pathways, visualizing tumor responses, and understanding the implications of different therapeutic choices in a tangible, engaging way. Such advances would not only enhance clinical decision-making but also promote patient empowerment and shared decision processes.

Moreover, ongoing improvements in wearable and implantable sensor technologies will enrich the data fed into DTs, enabling even more granular and continuous monitoring of physiological parameters, treatment adherence, and lifestyle factors. As sensors become more sophisticated and less invasive, they will facilitate a truly holistic, real-time understanding of each patient’s health status.

Finally, advances in AI, particularly in the areas of explainable AI and federated learning, will be fundamental to ensuring that DTs remain transparent, interpretable, and secure. These developments will help address current concerns about the “black box” nature of AI, fostering greater trust among clinicians and patients and supporting regulatory approval.

### Barriers and challenges to implementation

Despite the promise of DT technologies in modifying PCa care, their translation from conceptual innovation to routine clinical application is fraught with substantial challenges. These barriers are multifaceted, spanning technical, regulatory, ethical, cost, and organizational dimensions, in addition to trials- and human acceptance-related issues.

The main barriers to the implementation of DT technologies in clinical practice, together with proposed strategic solutions, are presented in Table 4.

**Table 4:** Barriers and strategic solutions to implementing DTs in clinical practice

Barrier	Strategic solution
Data fragmentation	Interoperable platforms with standardized formats
Limited validation of DTs	Prospective clinical trials embedded in clinical workflows
Ethical/data privacy concerns	Robust GDPR-compliant governance and patient-centric transparency models
Computational complexity	Integration of quantum computing and federated learning pipelines
Low-resource settings infrastructure	Scalable, cloud-based DT models with reduced hardware footprint
Limited clinical validation of benefit	Prospective trials demonstrating outcome improvement and cost-effectiveness
Physician and patient acceptance	User-centered design, transparency, explainable models, training, and shared decision-making

*DT* Digital twin*, GDPR* General data protection regulation

A major technical obstacle lies in the integration and harmonization of highly heterogeneous data sources. PCa management requires the assimilation of clinical records, imaging data, genomic and proteomic information, and even real-time physiological signals from wearable devices. Each of these data streams comes with its own standards, formats, and degrees of reliability. Establishing a unified, interoperable framework that can effectively handle this complexity without compromising data quality or interpretability represents a daunting task.

Moreover, the development of robust, transparent, and explainable AI algorithms is essential. Clinicians and patients alike need to understand and trust the outputs of a DT, particularly when these outputs influence critical medical decisions. The mentioned “black box” nature of many AI models remains a significant concern, underscoring the importance of creating systems that are not only accurate but also interpretable and auditable.

From a regulatory standpoint, DT technologies challenge existing frameworks governing medical devices and decision-support systems. Regulatory bodies must establish clear guidelines for validating and certifying DT-based tools, ensuring they meet stringent safety and efficacy standards before they can be integrated into patient care. The pathway to obtaining necessary approvals, such as European conformity–CE marking in Europe or Food and Drug Administration–FDA clearance in the United States, can be long and resource-intensive, potentially delaying widespread adoption.

Ethical and privacy considerations present further hurdles. The creation and continuous updating of a DT necessitate the collection and processing of vast amounts of sensitive personal health data. Ensuring patient consent, data security, and compliance with privacy regulations such as the General Data Protection Regulation (GDPR and the AI Act is imperative. Additionally, there are ethical questions surrounding data ownership and the potential for algorithmic biases, which could inadvertently exacerbate existing healthcare disparities if not properly addressed.

On an organizational level, integrating DT technology into established clinical workflows demands substantial cultural and infrastructural shifts. Healthcare professionals must be adequately trained to interpret and utilize DT outputs effectively, and institutional support is needed to invest in the necessary computational infrastructure. Resistance to change, particularly in resource-constrained settings, can hinder the adoption of these technologies.

Finally, economic factors cannot be overlooked. The development, deployment, and maintenance of DT systems require significant financial investment. These costs primarily derive from the need for high-performance computing resources, secure interoperable data platforms, and specialized technical expertise for data engineering, model development, and maintenance. Similar to advanced imaging technologies such as MRI, these infrastructural and organizational requirements may limit widespread implementation, particularly in resource-constrained settings. Nevertheless, DT ecosystems are inherently software-based and modular; once developed, they may be deployed through scalable, cloud-based architectures and shared computational resources, potentially lowering marginal costs and facilitating adoption even in low- and middle-income countries, where they would necessarily rely on a minimal core dataset, thereby helping to reduce global disparities in cancer care. Indeed, most DT components rely on software infrastructure rather than dedicated hardware installations, cloud-based or federated implementations may allow resource sharing across institutions.

Demonstrating a clear return on investment, both in terms of improved patient outcomes and cost savings for healthcare systems, will be crucial for garnering support from payers and policymakers. Finally, beyond technical and infrastructural constraints, broader translational challenges must also be considered. The clinical value of DT systems must be demonstrated through prospective studies showing meaningful improvements in outcomes, cost-effectiveness, or workflow efficiency. Moreover, adoption depends on physician trust and willingness to integrate algorithmic decision-support into practice, as well as patient acceptance of data-driven recommendations. Without demonstrable clinical benefit and adequate user confidence, even technically robust DT platforms may fail to achieve routine implementation. Addressing these barriers will require a coordinated, multidisciplinary effort involving clinicians, data scientists, engineers, ethicists, regulators, and patient advocacy groups. Only through such collaboration can the full potential of DT technologies in PCa, and more broadly in precision medicine, be realized.

## Conclusions

The emergence of DT technologies represents a paradigm shift in the management of complex diseases such as PCa. By creating dynamic, data-driven virtual representations of individual patients, DTs offer unprecedented opportunities for personalized care, continuous monitoring, and proactive intervention. As we move towards an era of increasingly individualized medicine, the integration of DTs into clinical workflows promises to enhance outcomes, and empower patients.

## Data Availability

Not applicable.

## Abbreviations

AI: Artificial intelligence
DT: Digital twin
MicroRNA: Microribonucleic acid
mpMRI: Multiparametric magnetic resonance imaging
PCa: Prostate cancer
PI-RADS: Prostate imaging-reporting and data system
PSA: Prostate-specific antigen
